# Deformities: A Treatise on Orthopædic Surgery

**Published:** 1897-09

**Authors:** 


**Deformities: A Treatise on Orthopædic Surgery.** By A. H. Tubby, M.S. Pp. xxii., 598. London: Macmillan and Co., Ltd. 1896.

The National Orthopædic Hospital and the Evelina Hospital have supplied Mr. Tubby with most of the opportunities which rendered this volume possible, and while it is obviously drawn chiefly from the writer's own experience, it also bears evidence of careful study of the work of others, particularly of Bradford and Lovett, in America; of Rédard, in France; of Hoffa, in Germany; and of Adams, Reeves, Walsham and Hughes, in this country.

The major portion of the book is naturally devoted to the subjects of deformities of the spine and the various forms of club-foot, and a careful perusal of what the author says on these important subjects has given us much profit and pleasure. In the treatment of spinal caries we are glad to notice that no fanciful or complicated apparatus is recommended. Plaster-of-Paris jackets are spoken of with approval, and judicious remarks are made as to their proper application; while special attention is devoted to some other forms of apparatus for this affection, such as poro-plastic jackets and Taylor's brace. The operation of laminectomy for compression paraplegia is only briefly described, but references are made to other authorities for fuller particulars. Scoliosis is very fully dealt with, but we are rather surprised that massage in this affection is apparently so lightly thought of as to be hardly mentioned. The author seems to rely almost entirely on gymnastic exercises with intervals of rest in the recumbent posture. In the treatment of congenital talipes the author favours the long-established London practice of free division of tendons. Thus, in the second degree of congenital club-foot he recommends the division of the plantar fascia, the tibialis anticus and posticus, the flexor longus digitorum, perhaps the extensor proprius pollicis, and certainly the tendo Achillis. Macewen showed long ago that all this tenotomy was unnecessary, and only had the effect of weakening the foot. The deformity is due, not to a mere contraction of the tendons, but to a malformation of all the structures of the foot, including the bones and ligaments, which latter are far more unyielding than the muscles and tendons, so that, with the single exception of the tendo Achillis, nothing is to be gained and everything to be ost by cutting tendons.

The chapters on knock-knee, torticollis, webbed fingers, and other allied subjects are excellent, and are fully illustrated from the author's clinical practice.

The book is written in a pleasant style, is very fully illustrated, and forms a most trustworthy guide to the subjects of which it treats.

